# *LongQt*: A cardiac electrophysiology simulation platform

**DOI:** 10.1016/j.mex.2016.11.002

**Published:** 2016-11-28

**Authors:** Birce Onal, Daniel Gratz, Thomas Hund

**Affiliations:** a The Dorothy M. Davis Heart and Lung Research Institute, The Ohio State University Wexner Medical Center, Columbus, OH, USA; b Department of Biomedical Engineering, College of Engineering, The Ohio State University, Columbus, OH, USA; c Department of Internal Medicine, The Ohio State University Wexner Medical Center, Columbus, OH, USA

**Keywords:** LongQt, cardiac arrhythmia, action potential, ion channels, mathematical modeling, computer simulation

## Abstract

Mathematical modeling has been used for over half a century to advance our understanding of cardiac electrophysiology and arrhythmia mechanisms. Notably, computational studies using mathematical models of the cardiac action potential (AP) have provided important insight into the fundamental nature of cell excitability, mechanisms underlying both acquired and inherited arrhythmia, and potential therapies. Ultimately, an approach that tightly integrates mathematical modeling and experimental techniques has great potential to accelerate discovery. Despite the increasing acceptance of mathematical modeling as a powerful tool in cardiac electrophysiology research, there remain significant barriers to its more widespread use in the field, due in part to the increasing complexity of models and growing need for specialization. To help bridge the gap between experimental and theoretical worlds that stands as a barrier to transformational breakthroughs, we present *LongQt,* which has the following key features:

• Cross-platform, threaded application with accessible graphical user interface.
• Facilitates advanced computational cardiac electrophysiology and arrhythmia studies.
• Does not require advanced programming skills.

## Graphical abstract

**Figure:**
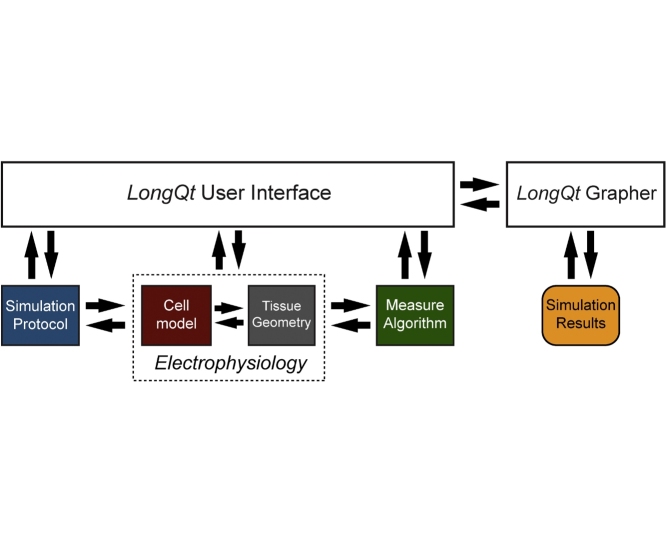

## Method details

### Structure of LongQt simulation software

*LongQt* simulation software utilizes the *Qt* application framework (version 5.6 or later found at https://www.qt.io/), and may be compiled to run on Mac (version OS X), Windows (version 7), or Linux systems. *Qt* is built on the standard C++ programming language and has bindings for programming in languages such as C++, Python, Java, or C#. Static compiled versions for PC or Mac and link to source code on Github are available under the “Research” section at http://hundlab.org. *LongQt* uses an open-source object-oriented design where cell geometry and electrophysiology are defined in a “Cell” class (parent class for cell-specific classes) and information pertaining to the specific simulation protocol is placed in a “Protocol” class. Additional classes are included to support multicellular simulations (“Fiber” and “Grid”), a graphical user interface, and graphing utility. *LongQt* allows for simulation customization through manipulation of these objects without composing *de novo* code or re-compiling. *LongQt* offers a wide range of utilities for simulating and analyzing model behavior, including the ability to: 1) track the time dependence of any state variable [e.g. transmembrane potential (*V_m_*)]; 2) measure important properties related to state variables (e.g. duration in time, amplitude, max or min value); and 3) parameter sensitivity analysis. *LongQt* automatically saves simulation output as labeled text files in a timestamped folder at the end of each simulation. Specified data may also be visualized in the *LongQt* user interface at the end of the simulation, although it is recommended that raw data files be used to generate publication ready figures offline. *LongQt Grapher* is a supplementary visualization program that may be used to view saved results without having to run a simulation.

### Mathematical models available in LongQt to simulate the cardiac action potential

Growing interest in computer simulation as a valuable tool for generating and testing hypotheses has yielded a large number of physiological mathematical models of action potentials from different cardiac cell types and species [1], [2]. *LongQt* offers the user the option of selecting from several different published mathematical models developed by our lab and others [3], [4], [5], [6]. These mathematical models all use the following general equation to describe the time dependent change in transmembrane potential:

$$C_{m}\frac{\partial V_{m}}{\partial t}=-\sum I_{ion}+I_{stim}$$

Here *C*_m_ represents the membrane capacitance, *I*_ion_ represents the total transmembrane current (carried by distinct populations of ion channels), *I*_stim_ represents the external stimulus current, and *V_m_* represents the transmembrane potential. *LongQt* uses the forward Euler method with a dynamic time step to solve the governing set of ordinary differential equations for each model. For multi-cellular simulations, electrical propagation is described using the cable equation, a linear parabolic partial differential equation (PDE):

$$\frac{d}{4R_{i}}\frac{\partial^{2}V_{m}\left(x,t\right)}{dx^{2}}=C_{m}\frac{\partial V_{m}\left(x,t\right)}{dt}+\sum I_{ion}$$

Where *d* represents the fiber diameter, *R_i_* represents the intracellular resistivity of the cell, *V_m_* represents the membrane voltage, *C_m_* represents the membrane capacitance, and *I*_ion_ represents the ionic current. For one-dimensional simulations, propagation along the discretized fiber is solved numerically using the Crank-Nicolson implicit method. For two-dimensional tissue simulations, the PDE is solved using the Peaceman-Rachford method of alternating implicit solutions for rows and columns [6], [7], [8].

*LongQt* allows the user to select action potential (AP) models corresponding to several different cardiac myocyte types (atrial, ventricular, and sinoatrial) and/or species (human, rabbit, and dog) [3], [4], [5], [6]. Specifically, users may implement action AP models of the canine (normal or diseased) [6] or human ventricular myocyte [3], human atrial myocyte [4], or rabbit central sinoatrial node (SAN) myocytes [5]. Additional models have been programmed in C++, are compatible with *LongQt*, and will be incorporated into future versions of *LongQt* [9], [10], [11], [12], [13], [14]. Each model is uniquely suited to help answer different questions relevant to cardiac electrophysiology and arrhythmia. Simulation of the Hund-Rudy (HRd) dynamic model using *LongQt* is useful for researchers interested in the Ca^2+^/calmodulin-dependent protein kinase (CaMKII) regulatory pathway and rate-dependent changes in AP behavior in normal and diseased myocytes (epicardial canine infarct border zone) [6]. The human atrial model of Grandi et al. available in *LongQt* has been used to study AP remodeling and atrial fibrillation [4]. The rabbit sinoatrial node mathematical model developed by Kurata et al. is notable for simulating *V_m_* and Ca^2+^ concentration changes in the central region of the rabbit SAN [5]. Finally, *LongQt* provides access to the widely used and well-validated model of the human ventricular myocyte developed by Ten Tusscher *et al.* [3]. Users of *LongQt* are referred to the original publications for details on a specific model.

### Use of control panels in LongQt

**Fig. 1:**
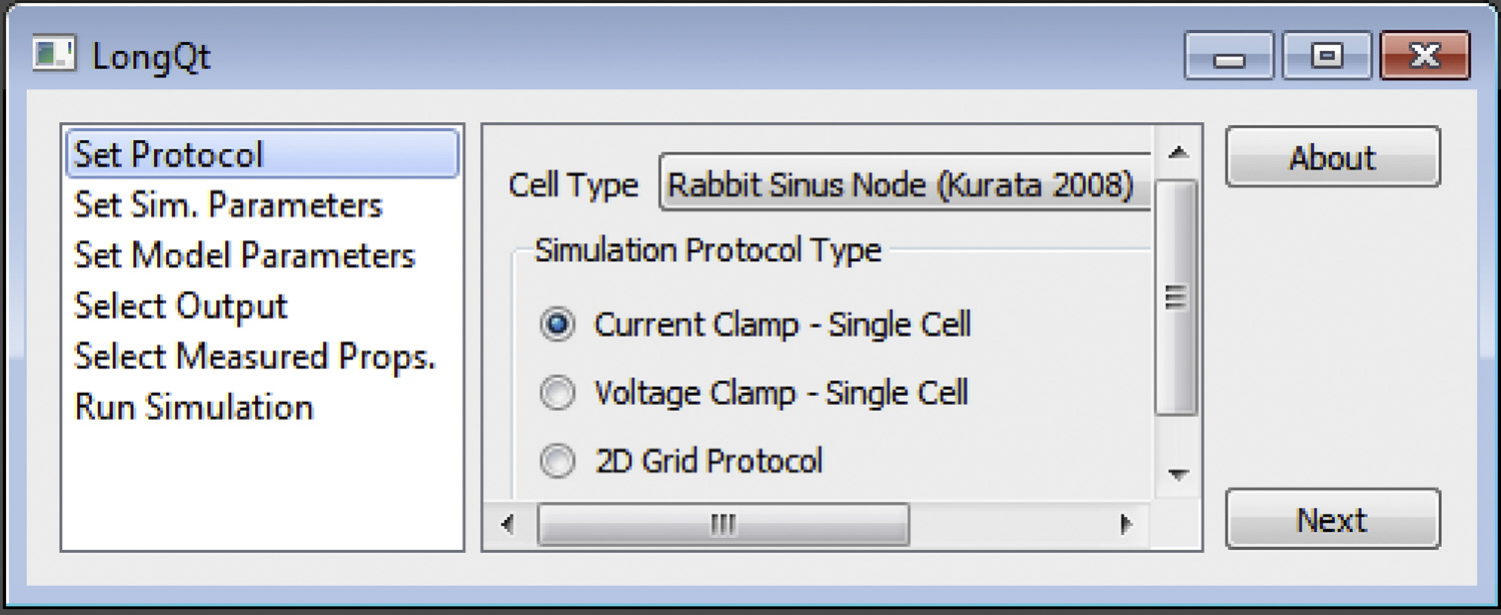
Initial *LongQt* interface upon launching the program. Users may navigate different panels on the left-hand side control panel to customize their simulation.

Simulations are designed and executed from the *LongQt* simulation interface by navigating through a series of menus (Fig. 1). The sidebar on the left-hand side of the interface lists the available control panels in the recommended order of completion (although it is possible to jump between panels): 1) “Set Protocol”; 2) “Set Sim. Parameters”; 3) “Set Model Parameters”; 4) “Select Output”; 5) “Select Measured Props”; and 6) “Run Simulation” (Fig. 1).

### Selecting the simulation mode

The initial screen (“Set Protocol”) gives the user the option of selecting from three different simulation modes: 1) single-cell current clamp for studying the AP response to current stimulation; 2) single-cell voltage clamp for simulating transmembrane current response to voltage pulse stimulation; and 3) multi-cellular fiber (one-dimensional geometry) or grid (two-dimensional geometry) to study electrical impulse propagation. The user may also select the AP model cell type using a dropdown screen in the upper right corner of the menu. Once a mode has been selected, the user may advance through the rest of the panels to select parameters tailored for that specific simulation mode. Menus automatically populate with abbreviated variable/parameter names; a complete description may be found by hovering over the variable of interest.

### Defining a simulation protocol

**Fig. 2:**
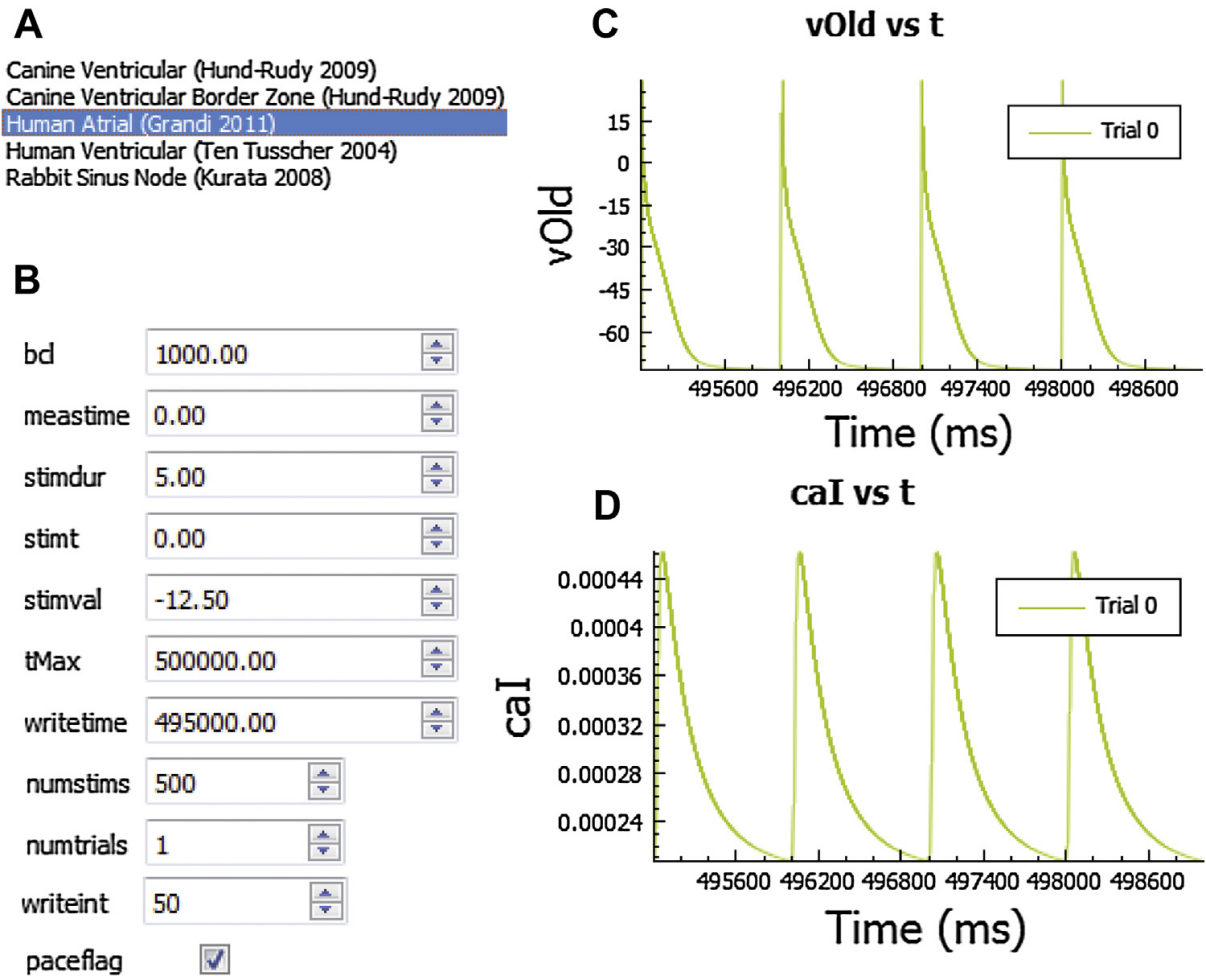
Overview of current-clamp simulation mode in *LongQt.* (A) List of available cardiac action potential mathematical models for simulation. (B) Example of “Set Sim. Parameters” panel for current-clamp protocol. Representative (C) action potential (*vOld*) and (D) intracellular calcium concentration (*caI*) for a Grandi atrial model^4^ current clamp simulation, using simulation parameters specified in B. Abbreviations are as follows: *bcl*, basic pacing cycle length in milliseconds (ms); *meastime*, time to begin measuring variables (ms); *stimdur*, the duration of injected stimulus current (ms); *stimt*, time to inject the first stimulus current (ms); *stimval*, the amplitude of injected stimulus current in μA/μF; *tMax*, the maximum duration of the stimulation (ms); *writetime*, time to begin writing/analyzing model variables (ms), *numstims*, the total number of pacing stimuli to apply; *numtrials*, the total number of independent simulations to perform; *writeint*, indicates sampling data interval for variables in select output; *paceflag*, checked to apply pulse train.

**Fig. 3:**
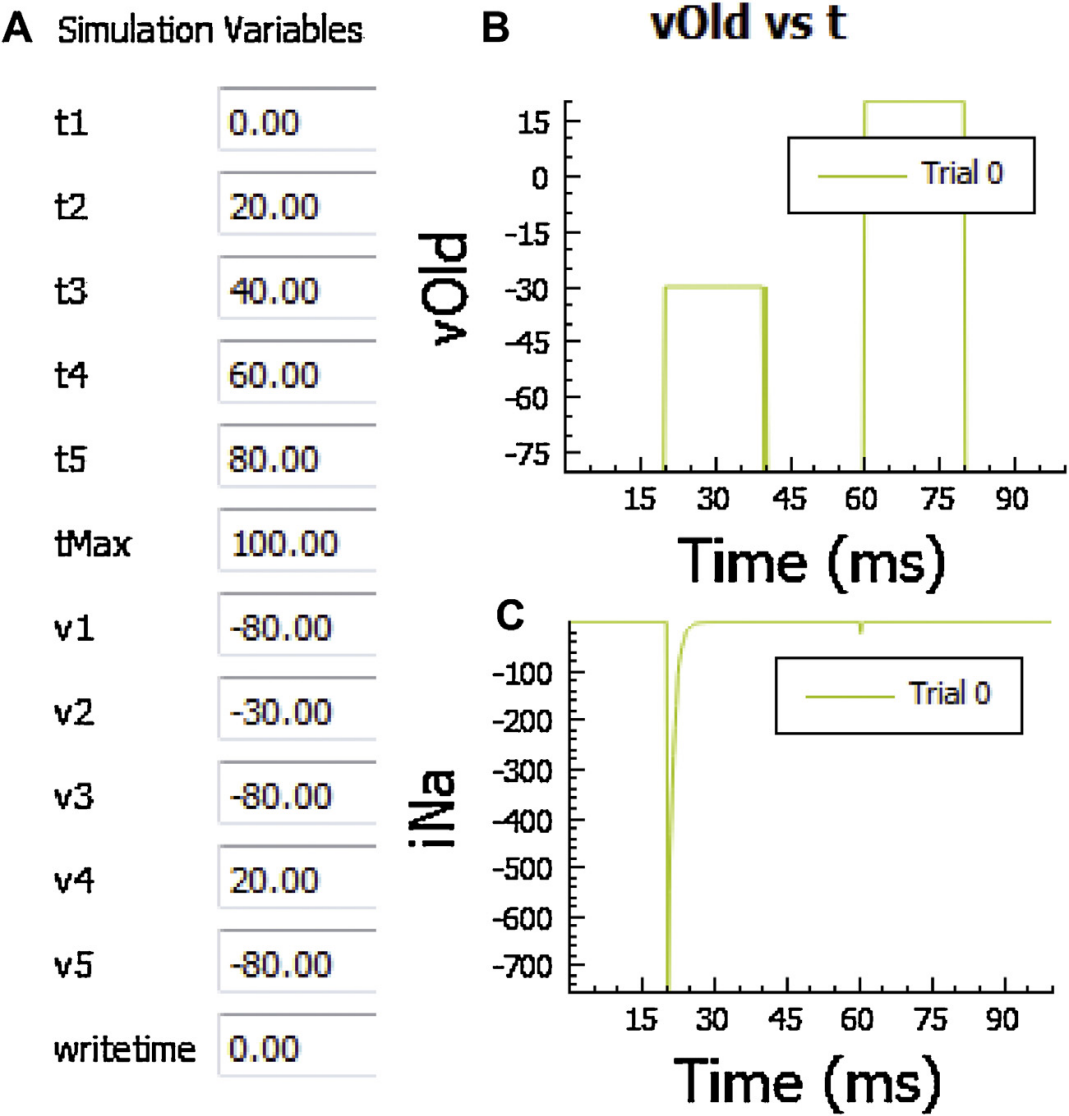
Overview of voltage-clamp simulation mode in *LongQt*. (A) Example of “Set Sim. Parameters” panel for voltage-clamp protocol. Representative (B) transmembrane potential (*vOld*) and **(C)** sodium current (*iNa*) vs. time for a Grandi Atrial model voltage clamp simulation, with simulation parameters specified in A. Abbreviations are as follows: *t1-t5*, timepoints at which voltage is changed (ms); *v1-v5*, transmembrane potential corresponding to timepoints *t1-t5* (mV); *tMax*, maximum duration of simulation (ms); *writetime*, time to begin writing/analyzing model variables (ms).

In the “Set Sim Parameters” panel, the user may customize a simulation protocol by specifying parameter values related to the stimulus, times for measuring/outputting data, and simulation duration (Fig. 2). For example, in current clamp mode (specified in “Set Protocol” menu described above), a current pulse train may be applied by defining the basic pacing cycle length, current stimulus duration and amplitude (*bcl, stimdur* and *stimval* respectively), total number of stimuli and time to apply first pulse (*numstims* and *stimt*, respectively), and other relevant parameters. Default values for all parameters (including model-specific values for stimulus amplitude/duration) are automatically loaded into *LongQt* (default is a pulse train of 500 stimuli). A default stimulus current is provided for all models, including the sinus node model (for the purpose of phase resetting or cycle length restitution studies [15]). All models account for the conservation principle through the external stimulus current, and do not incur drift in intracellular ion concentrations for long-term pacing studies [16]. In voltage-clamp mode, the “Set Sim. Parameters” panel provides the user with the ability to define a voltage pulse protocol by adjusting the time and corresponding *V_m_* clamp values (Fig. 3). Specifically, the user may specify 5 different values (*v1*–*v5*) and corresponding timepoints (*t1*–t*5*) for clamping *V_m_* (Fig. 3A). As with current clamp studies, time profiles of *V_m_* and selected model variables (e.g. transmembrane current) are available for visualization (Figs. 3B and C).

**Fig. 4:**
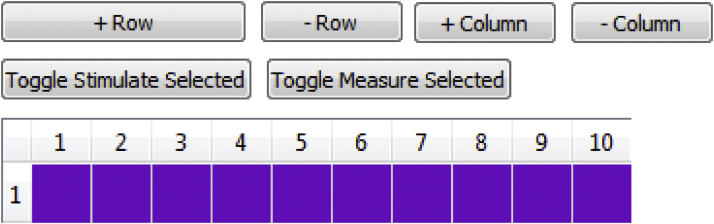
Multi-cellular simulation protocol. In order to set up a multicellular grid, the user adds or subtracts rows and columns of connected cells by navigating the top buttons (*+Row, −Row, +Column, −Column*). Ten cells connected horizontally are shown here. The user may specify the mathematical model for each node and whether or not to stimulate/record data by selecting nodes/rows/columns (click and drag may be used to specify a region.). On Windows platforms, the row or column numbers of selected regions become bold (number bold does not happen on Mac, but user may still click on a region and specify/toggle node properties).

In addition to single cell studies, it is often desirable to analyze electrical impulse propagation and arrhythmia mechanisms in cardiac tissue. *LongQt* provides support for multicellular simulations with a control panel that allows for definition of one- or two-dimensional geometries (Fig. 4). This mode allows the user to simulate propagation in a homogeneous or heterogeneous fiber/grid. Users may customize tissue geometry by adding columns or rows of nodes and by specifying the cell model type at each node indicated by node color (Fig. 4). By selecting a node/row/column (click and drag may be used to select a region), the user may define which nodes will be stimulated and/or monitored for variable properties (e.g. AP duration, Ca^2+^ transient amplitude).

**Fig. 5:**
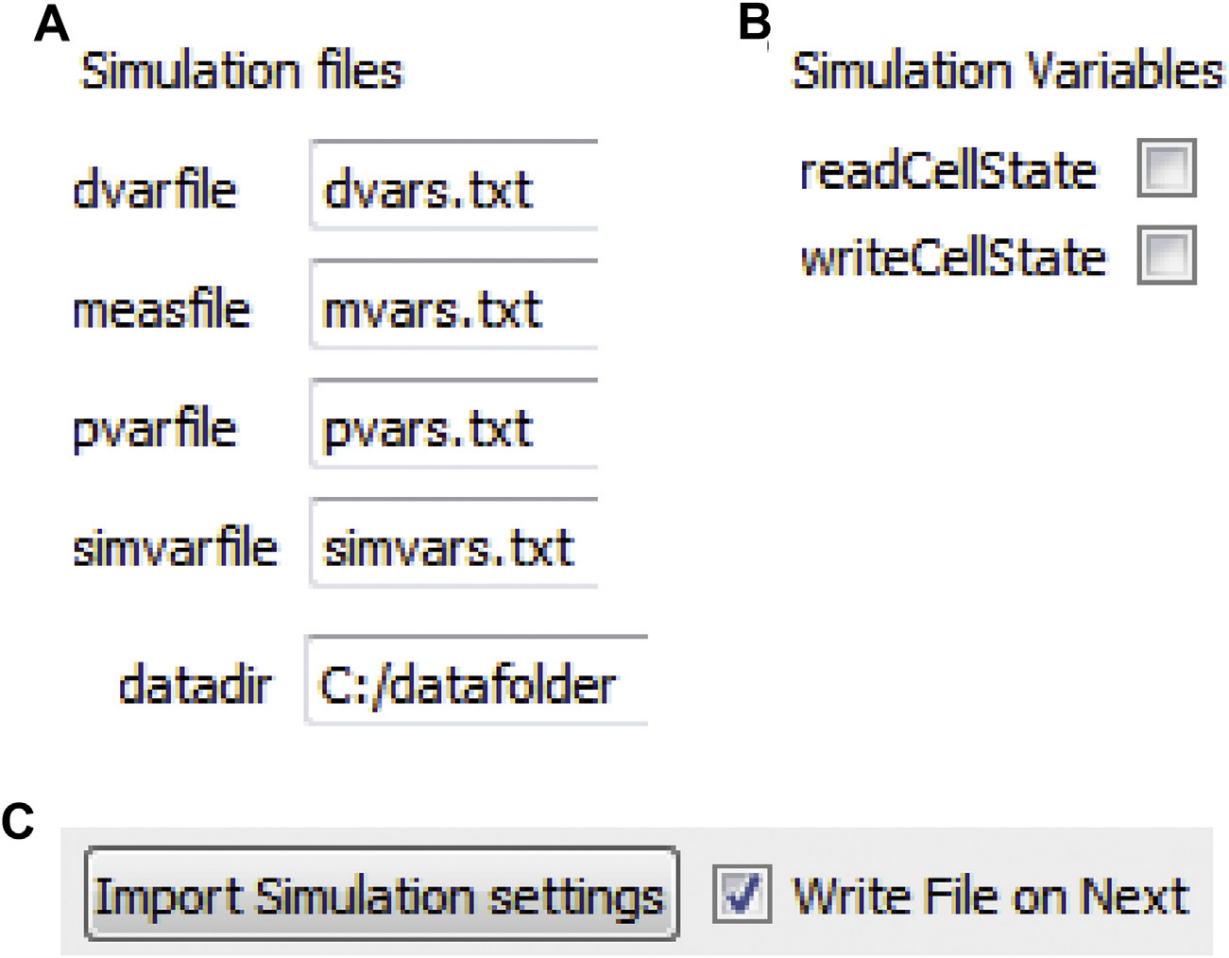
Reading, writing data and importing settings in the “Set Sim. Parameters” panel. (A) The user may define filenames where *LongQt* will save simulation settings (*dvars*, *mvars, pvars, simvars*), and the directory where all files will be saved. (B) The user may choose to input initial conditions from another simulation (*readCellState*) or output conditions to a file at the end of the simulation (*writeCellState*). (C) The user may set all simulation conditions to those from a previous simulation by clicking “Import Simulation settings” and selecting existing files (*dvars, mvars, pvars, simvars*) from another folder. When the user hits the “next” button for each panel, a “.txt” file of the items selected in the current panel is generated automatically if “Write File on Next” is checked.

The “Set Sim. Parameters” panel includes a “Simulation files” tab that specifies file names and directories for the simulation. In order to cross-reference output data with input simulation details, the settings for each simulation are all saved in the same directory as the output data of the simulation. The parameters for one simulation are saved in four different files: *simvars* for parameters that control the simulation indicated in “Set Sim. Parameters” (such as simulation time, pacing frequency); *pvars* for parameters related to model parameters/parameter sensitivity analysis (indicated in “Set Model Parameters”); *dvars* for output variables indicated in “Select Output”; and *mvars* for variables that are being measured indicated in “Select Measured Props”. These four files, along with any raw output data from the simulation, are saved in a timestamped data directory for every simulation, in addition to a text file of notes the user wished to include with the simulation. If multiple trials are run at the same time, the input and output files are all saved in this directory with the trial number specified in the file names. Thus, a complete log of model variables and parameters used to generate a set of results are automatically generated for each simulation. The names of these files and the directory can be changed by selecting the “Simulation files” tab in the “Set Sim. Parameters” panel (Fig. 5A). At the end of the simulation, a time-stamped panel appears as an additional selection in the master control panel of the interface, which allows the user to view time profiles of *V_m_* and any other state variables (up to 10) selected in the “Select Output” panel (discussed in more detail below) prior to running the simulation (Figs. 2C and D).

Initial conditions for state variables may be read into any simulation by selecting “readCellState” from the “Set Sim. parameters” panel (Fig. 5B) (otherwise, model will use default resting steady state conditions). This feature allows a user to select a specific set of initial conditions from a folder containing the initialization file (*dss0_cellState.txt*, specified in the “Simulation files” tab), which specifies the name and initial values of all model variables required to uniquely define the state of the system. Alternatively, the user can copy *dss0_cellState.txt* into the current working directory. If the user wants to save final model variables from a simulation to use as initial conditions for a subsequent simulation the user can check “writeCellState” (Fig. 5B), and values are written to *dss0_cellState.txt* in the output directory folder (*datadir*) specified in the “Simulation files” tab. *LongQt* also allows the user to import all settings corresponding to a previous simulation by clicking on “Import Simulation settings” (Fig. 5C) in the corresponding panel (e.g. to import a previously generated simvars file the user would click “Import Simulation Settings” in the “Set Sim. Parameters” panel), which implements any *simvars*, *dvars*, *mvars*, or *pvars* file from a previously generated data directory.

### Altering action potential model parameters

*LongQt* allows the user to modify select cell model parameters (mostly ion channel conductances) without having to recompile the code using the “Set Model Parameters” panel. To do so, first select a parameter of interest from the pulldown menu and click the “+” to add it to the list (click “-‘ to remove a selection from the list). An extended definition may be found by hovering over the parameter name. Once a parameter has been added to the list, its value may be specified three different ways using adjacent pulldown menus: 1) set to a single parameter value using “init value’ and selecting specifying a number (in most cases, the value would represent a multiplicative factor of the default value); 2) Iterate parameter from an initial value by a fixed increment (multiple trials, select ‘iter’ and specify initial value and increment for each trial; should be used with numtrials >1 specified in “Set Sim Parameters,” otherwise simulation will just use initial value); and 3) Specify parameter to a random value (normal or lognormal distribution with adjustable mean, standard deviation, may be used with single or multiple trials). Running multiple trials with random selection of parameter values is an easy way to perform model sensitivity analysis [17], [18]. *LongQt* utilizes the threading utility afforded by *Qt* to allow for concurrent execution of multiple trials.

### Data output

In the “Select Output” panel the user selects which model-specific state variables to record over time (e.g. intracellular ion concentrations, transmembrane currents, gating variables). Time and transmembrane potential (*V_m_*) are automatically selected for every simulation output. The sampling data interval for the values chosen in the “Select Output” panel can be changed in the previous panel, “Set Sim Parameters” by changing the value of ‘writeint’ (Fig. 2B). *LongQt* also employs an adaptive timestep for all models in order to improve computational efficiency; this timestep is described for each model in the table below.

**Table:** 

Model	Dtmin (ms)	Dtmed (ms)	Dtmax (ms)	Dvdtmax (ms)	dvcut (ms)
Grandi	0.005	0.005	0.005	5.43E-10	1.0
Ten Tusscher	0.005	0.01	0.1	7.29E-7	1.0
Hund-Rudy	0.005	0.01	0.1	5.43E-10	1.0
Kurata	0.005	0.05	0.05	–	1.0

**Fig. 6:**
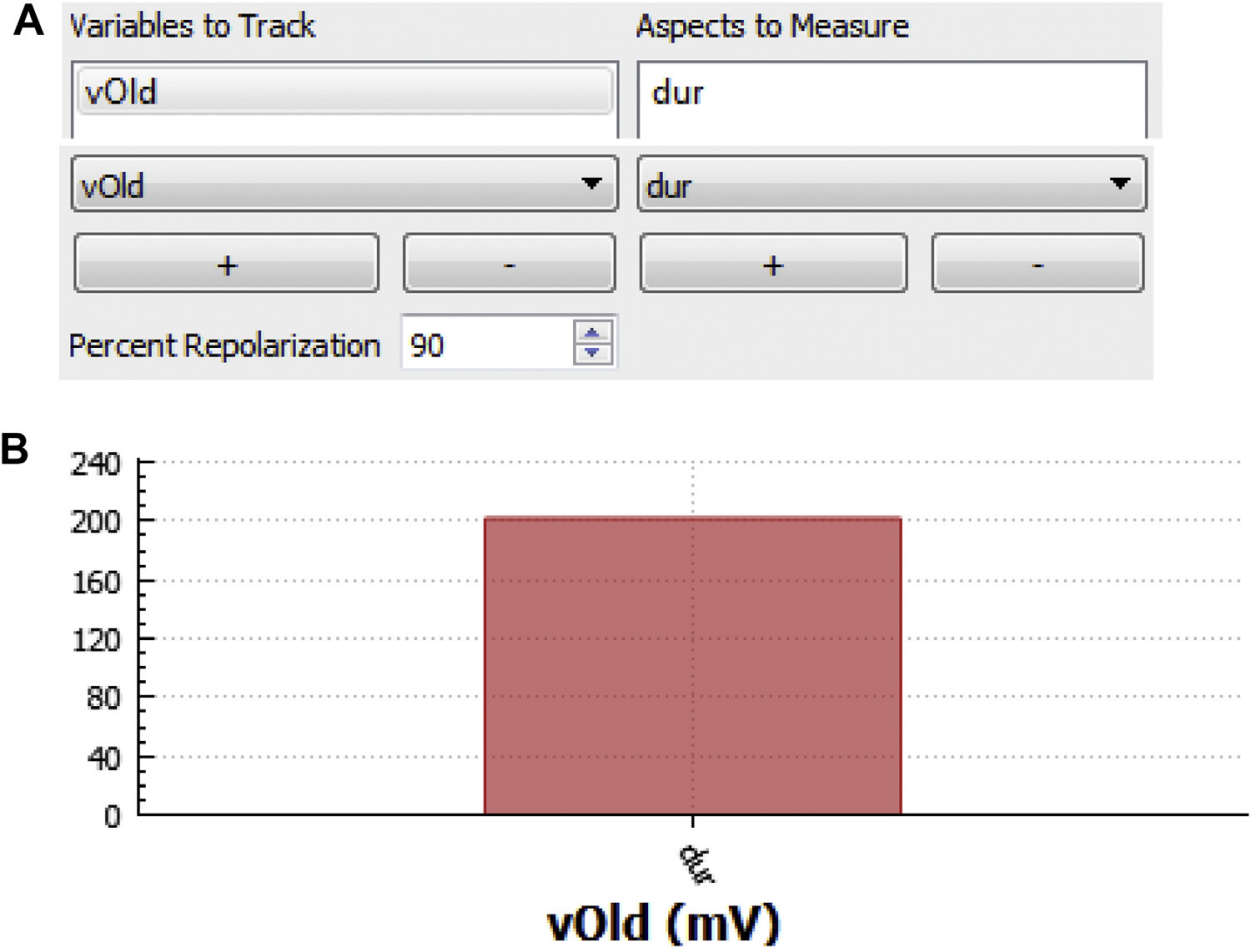
Overview of **“**Select Measure Props” panel. (A) In this panel the user may select a model variable [e.g. L-type Ca^2+^ current (*iCal*), *V_m_*, intracellular Ca^2+^) and property [e.g. minimal value (*min*), duration (*dur*)] to measure using built-in algorithms. (B) *LongQt* automatically graphs outputs from this panel at the end of the simulation (*red* *bar* indicates *dur* for *V_m_* for the current simulation). User may load in value(s) from previous simulation for comparison.

In the “Select Measured Props” panel the user has the option of tracking properties associated with state variables (Fig. 6). For example, minimal value, peak value, amplitude, and 90% duration may be determined for *V_m_*. Peak value can be determined for ion concentrations or any state variable of interest. Algorithms for measuring these values were optimized for *V_m_* or bulk Ca^2+^ concentration (full measure utility is only available for *V_m_* or bulk *Ca^2+^*) but peak value can be determined for any state.

### Executing the simulation

Finally, once desired settings have been entered, the user may launch the simulation from the “Run Simulation” panel. When the simulation completes, *LongQt* will show a completed progress bar and indicate the timestamped directory where all output data files were saved. This directory also includes the parameters the user chose in each panel to run the simulation.

### Analyzing and graphing simulation results

**Fig. 7:**
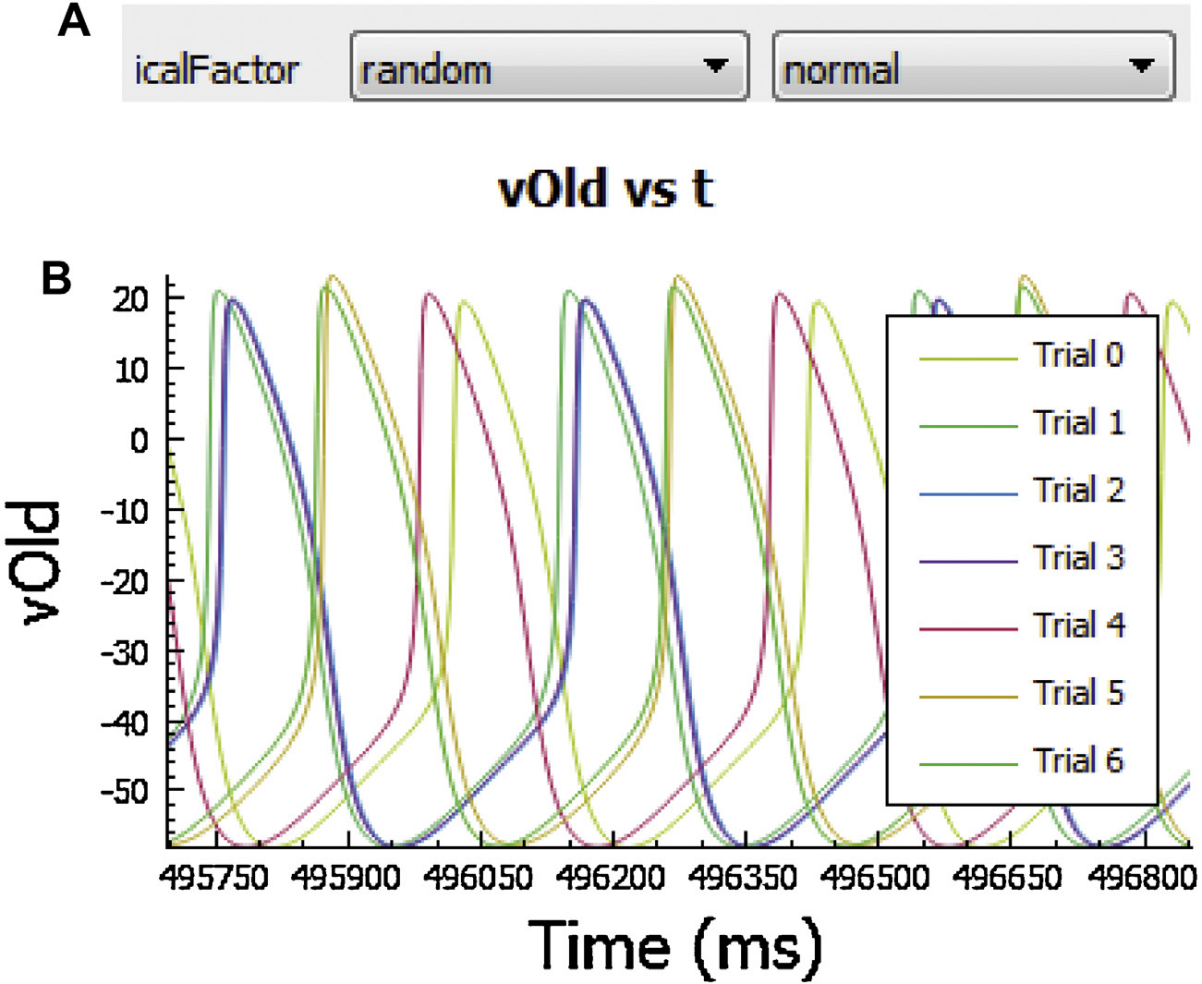
Overview of **“**Set Model Parameters” panel. In this panel the user may alter individual parameters in the action potential model (e.g. ion channel conductances). (A) Example of a simulation that scales the L-type calcium channel conductance using a normally distributed random scaling factor (*icalFactor*). (B) *LongQt* automatically graphs outputs from this panel at the end of the simulation. Action potential outputs of the Kurata sinoatrial node model with different L-type calcium channel conductance values are shown [4].

At the end of each simulation, a tab will appear in the left-hand side bar with the name of the timestamped folder with all saved output data. All variables selected in the “Select output” panel will be graphed as a function of time in the user interface at the end of the simulation (example graphs shown in Figs. 2, 3 and 6). Variables chosen in the “Select Measured Props” panel will be reported in a bar graph at the end of the simulation. Up to 10 choices can be visualized in the user interface, with the upper tabs marked with variable names. All graphs can be saved from the user interface for later use. Variables are also saved in a tab-delimited text file (example: *dt0_dvars.txt* will have columns with time and state variables for the first simulation) to allow for import into a more advanced graphing program and subsequent analysis. Previous simulations may also be imported directly into the graphing interface (Fig. 7B) to compare against the current simulation by clicking “Import Control” and selecting a previous data file. Users may also view saved data without having to run a *de novo* simulation in *LongQt Grapher* (provided in all static compiled versions of *LongQt). LongQt Grapher* generates the same output graphs that the user would see at the end of the simulation originally run in *LongQt*. The grapher automatically generates axis titles with units and allows for x-axis or y-axis zoom.

## Conclusions

The growth in power and availability of computers has increased access to high-performance modeling and simulation, but it remains inaccessible to biomedical researchers with limited programming experience. Increased availability of mathematical models through projects such as CellML, and increased access to high computing power has increased global support of mathematical modeling [2], [19], [20]. However, increasing complexity of models and programming expertise required to use these models is still a research barrier. A number of simulation platforms and model repositories have been developed to facilitate electrophysiology computer simulation and visualization, including OpenCOR, CESE Plus 2.0, Myokit and more [19], [21], [22], [23]. However, there remains a great need to bridge experimental and theoretical worlds in electrophysiology research. With the help of computational biology, researchers are able to perform studies that are difficult or even impossible in animals (especially in human). Possible applications include modeling the effect of drugs, isolating and perturbing single ion currents, testing the impact of genetic changes to ion channel function, and more. Tightly integrated experimental and modeling approaches have the potential to accelerate our discovery of basic mechanism and potential therapies for cardiac disease and arrhythmia. *LongQt* strives to bridge a knowledge gap for biomedical researchers by presenting an accessible, powerful, cross-platform, computationally efficient user interface that does not require extensive programming knowledge. In simpler terms, this work attempts to answer the question: can mathematical modeling be made accessible without sacrificing utility?

The work presented here represents a beta version of *LongQt*, which we hope will be useful for many applications. However, we acknowledge several limitations of the tool, some of which we expect to address with future versions. Further developments to the interface include incorporating other higher order methods beyond Forward Euler to solve the governing ordinary differential equations. *LongQt* would also benefit from Markov model parameterization to allow for users to fit their own patch-clamping data and generate new ion channel models. We are also interested in developing a code editor for users to incorporate their own formulations into models to fit experimental data. Our hope is to eventually develop a platform so that users with minimal programming knowledge can incorporate their own formulations or other models, and build a model that satisfies their simulation needs. We believe that further development of software tools such as *LongQt* makes computational biology experiments more accessible and increases impact on biological studies, allowing us to understand more about cardiac cell excitability as well as pathophysiological cell activity.
